# The impact of itch symptoms in psoriasis: results from physician interviews and patient focus groups

**DOI:** 10.1186/1477-7525-7-62

**Published:** 2009-07-06

**Authors:** Denise Globe, Martha S Bayliss, David J Harrison

**Affiliations:** 1 Amgen Inc, One Amgen Center Drive, Thousand Oaks, CA, 91320 USA; 2 Mapi Values, 3rd Floor 133 Portland Street, Boston, MA 02114, USA

## Abstract

**Background:**

The objective of this qualitative study was to better understand the impact of psoriasis symptoms using a 3-part process: 1) develop a disease model for psoriasis to identify the most important concepts relevant to psoriasis patients; 2) conduct interviews with dermatologists to identify key areas of clinical concern; and 3) explore psoriasis patients' perceptions of the impact of psoriasis.

**Methods:**

A disease model was developed from a review of the published literature and later revised based on the findings of clinician interviews and patient focus groups. To confirm the clinical relevance of the concepts identified in the disease model, 5 dermatologists were selected and interviewed one-on-one. They were asked to rate major psoriasis symptoms according to importance and bothersomeness level to patients on separate scales of 1 to 10. Results of clinician interviews were used to develop interview guides for patient focus groups. To identify important domains of psoriasis, 39 patients participated in 5 separate concept elicitation focus groups. Four focus groups included patients with severe psoriasis (n = 31) and one included patients with mild psoriasis (n = 8). Patients were asked to describe their current psoriasis symptoms and to rate them on a scale of 1 to 10, according to importance, severity, and troublesomeness. An average mean rating was calculated for each symptom throughout all focus groups.

**Results:**

Clinicians most frequently mentioned itch (n = 5), psoriatic arthritis or "joint pains" (n = 4), flaking (n = 4), and pain (n = 3) as primary physical symptoms of psoriasis. Three clinicians gave a rating of 10 for the importance of itch; two clinicians gave ratings of 8 and 7 for importance. The majority of patients rated itch as the most important (31/39), most severe (31/39), and most troublesome (24/39) symptom and noted that itch negatively impacted daily activities (eg, concentration, sleep, ability to attend work or school), as well as emotions (eg, anxiety and embarrassment).

**Conclusion:**

These analyses suggest that itch is one of the most important symptoms of psoriasis, contributing to diminished health-related quality of life (HRQoL) in patients with both mild and severe disease.

## Background

Psoriasis is a chronic, systemic, inflammatory disease characterized by erythematous, scaly plaques that can itch and bleed. It has a prevalence of approximately 2.2% in the U.S. [1]. Psoriasis has a major impact on health-related quality of life (HRQoL) that is comparable to other major medical diseases such as cancer, arthritis, hypertension, heart disease, diabetes, and depression [2,3]. Dysregulation of the immune system (eg, T cells and cytokines), intrinsic epidermal components (eg, keratinocyte growth and differentiation factors), and environmental factors (eg, stress, cold weather, excessive alcohol intake, and some medications) contribute to the chronic, relapsing nature of the disease [4-6].

Psoriasis is a highly symptomatic disease. Symptoms of psoriasis include burning, stinging, inflammation, redness, itching, pain, scaling, and cracking of skin. Some symptoms (itch, soreness, pain, stinging) are included in the Dermatology Life Quality Index (DLQI), a 10-item questionnaire commonly used in clinical trials to assess HRQoL [7]. Although the DLQI is a good indicator of overall HRQoL and has been validated for use in patients with psoriasis [8], it does not assess all symptoms and does not provide a measure of symptom severity. Additional dermatology-specific and psoriasis-specific instruments include the Dermatology Quality of Life Scales (DQOLS), the Dermatology Specific Quality of Life (DSQL), Skindex, the Psoriasis Disability Index (PDI), and the Psoriasis Life Stress Inventory (PLSI) [9]. Other instruments used to assess HRQoL in dermatology clinical studies, the Short Form 36 health survey (SF-36) and the EuroQOL 5D (EQ-5D), are not specific for dermatologic diseases and do not provide information on severity, importance, or improvement of specific symptoms [8].

The objective of this qualitative study was to better understand the impact of psoriasis symptoms using a 3-part process: 1) develop a disease model for psoriasis to identify the most important concepts relevant to psoriasis patients; 2) conduct interviews with dermatologists to identify key areas of clinical concern; and 3) explore psoriasis patients' perceptions of the impact of psoriasis. Fulfillment of these objectives will satisfy the first component of the U.S. Food and Drug Administration (FDA) draft guidance on patient-reported outcome (PRO) development [10].

## Methods

### Disease model

The intent behind the disease model was to depict a broad overview of the issues encompassing the entire spectrum of psoriasis and to diagrammatically illustrate the interrelationships between these issues. Risk factors, symptoms, and treatments associated with psoriasis provided the concepts for the disease model, which was also intended to characterize the impact of psoriasis on patient functioning and well-being.

We developed a preliminary disease model from a review of the published literature pertaining to symptoms, severity, and impact of psoriasis. The preliminary disease model, based on information gathered in the literature search, was then revised based on physician and patient feedback.

We conducted the literature search using Medline^® ^and the Mapi Research Trust database (a collection of articles composed of more than 15,580 articles on health outcomes assessment focusing on HRQoL and PROs). The following keywords were used in the Medline^® ^search strategy: "psoriasis" or "dermatology" or "psoriatic nail dystrophy" or "plaque psoriasis" or "flexural psoriasis" or "guttate psoriasis" or "pustular psoriasis" or "nail psoriasis" or "erythrodermic psoriasis" or "immune-mediated psoriasis" or "eczema" or "dermatology" (43,451 results); "itch" or "pain" or "irritation" or "discomfort" or "inflammation" or "distress" or "soreness" (494,017 results); "patient-reported" or "quality of life" or "health-related quality of life" or "patient-reported outcomes" (89,385 results); "physical impacts" or "social impacts" or "emotional impacts" or "sexual impacts"; "clinician-reported" or "clinician-reported outcomes"; "questionnaire" or "patient-reported" or "instrument" or "scale" or "measure" (97 results). The search was limited to English language articles published after 1990.

### Physician Interviews

Five dermatologists with experience identifying, diagnosing, and treating patients with psoriasis were screened by Amgen Inc. for one-on-one interviews. All interviews were conducted over the phone by Mapi Values (Boston, MA) using an interview guide with open-ended and closed-ended questions.

Four of the clinicians had over 10 years of experience, and one clinician had 7 years of experience. Four of the clinicians practiced medicine in a research and teaching capacity, while the fifth clinician practiced in a teaching capacity. Two of the physicians worked in both inpatient and outpatient care settings, while the other 3 worked solely with an outpatient population. All participants stated that they were familiar with the following psoriasis treatment modalities: systemic therapies; phototherapy, laser-assisted phototherapy; biologic therapies; and all topical medications.

Each dermatologist was asked to describe his or her opinions and experience dealing with major psoriasis symptoms such as itch, pain, burning, and scaling. The clinicians rated the importance and level of bothersomeness of each symptom to their patients. They also evaluated the relevance of the symptoms to psoriasis, location on the body, severity, and treatment and management challenges.

All interviews were audio-recorded and transcribed verbatim. The Atlas.ti (Atlas.ti Scientific Software Development GmbH, Berlin, Germany) qualitative analysis software program was utilized to analyze the clinician interview transcriptions. A codebook was developed and reviewed by all members of the research team. Sentence and paragraph segments of the transcribed verbatim interviews were then coded by a trained researcher and reviewed by a senior team member. Statistics to quantify inter-rater reliability were not calculated, but any discrepancies in coding were resolved through a discussion and consensus-building process. We performed a stratification analysis to identify patterns, themes, and key concepts, providing descriptive statistics (frequency and distribution of quotes, rank, or concordance correlations for assessing the relative distance or concordance between categories). This analysis identified the constructs and domains most important to the clinicians and most salient to the experience of psoriasis and its symptoms. We used the results of these interviews to develop the interview guides for subsequent patient focus groups.

### Patient Focus Groups

We conducted concept elicitation focus group discussions to assess patients' experience with psoriasis in terms of symptoms of the condition and the impact on functioning and well-being. Participants for this study were identified through a commercial recruitment agency (Global Market Research Group, Murietta, CA), which enlisted general practitioners to help with patient recruitment following the approval of the study protocol by the Copernicus Group Independent Review Board (IRB). Recruiting clinicians provided patients with an information letter explaining study procedures, compensation, and right to withdraw from the study without penalty or change in medical care. We chose the general practitioner patient population because these patients were less likely to be receiving systemic therapy and more likely to be symptomatic, ensuring the participation of patients with the full breadth of severity of psoriasis.

Thirty-one patients with physician-confirmed severe psoriasis (≥ 10% body surface area [BSA] affected by psoriasis and Psoriasis Area and Severity Index [PASI] score ≥ 10) participated in 4 focus groups (6 to 9 patients per group). Eight patients with physician-confirmed mild psoriasis (≥ 3% BSA affected by psoriasis and PASI score = 3) participated in an additional focus group. The aim of the focus groups was to collect participants' experiences with psoriasis; specifically, information about psoriasis symptoms and the impacts of those symptoms on their lives.

During the concept elicitation phase of the focus groups, patients were asked to report the psoriasis symptoms they were currently experiencing and describe how psoriasis impacted their daily activities. Participants were also probed about several well-known psoriasis symptoms if these symptoms were not already mentioned by patients spontaneously. Following the discussions, patients were then asked to rate their current psoriasis symptoms on a visual analogue scale of 1 to 10, based on importance, severity, and troublesomeness (symptoms that were not currently experienced were rated 0). All symptoms, both spontaneous and probed, were rated by focus group participants. The average mean rating was calculated for the importance of each symptom throughout all focus groups and combined into an overall average importance score for each symptom.

Audio- and video-recordings were collected from all focus groups and transcribed by a transcription agency (Fantastic Transcripts, Boston, MA). The transcriptions were analyzed using Atlas.ti software. A codebook for patient focus groups, similar to the codebook that was devised for clinician interviews, was developed by the research team. The transcriptions from the focus groups were coded by a trained researcher and reviewed by a senior member of the team. Discussion and consensus-building were used to resolve coding discrepancies. We used descriptive statistics to characterize demographics, socioeconomic status, and disease characteristics of the focus group participants.

We developed a saturation grid for patient feedback in order to summarize the concept elicitation information found during the qualitative analysis. Conceptual saturation refers to the endpoint in the qualitative data collection and analysis process when further data collection and analysis cease to generate any new or distinctive categories, high level concepts, or substantive codes [11]. As opposed to quantitative analysis, in which an ideal sample size is calculated before data collection based on previous research and literature review, final sample size in qualitative research is driven by the prospective discovery of concepts, and hence there is no specific number that represents an "ideal" sample size [12].

We grouped the responses from each focus group by domain, sub-domain, and patient. The number of elicited responses were categorized by concept and reviewed across patients for each focus group. To construct the grid, the results for each concept from one focus group were compared to the results from a second focus group, based on the constant comparative method [13]. The totals for each concept from the first two focus groups were then compared to the third focus group. Next, the totals for each concept from the first three focus groups were compared to the fourth focus group. Finally, the totals from the first four focus groups for each concept were compared to the fifth focus group totals. Saturation is reached when subsequent focus groups or interviews do not yield any new information or concepts. The sample size of 39 patients was deemed to be adequate to achieve saturation based on established guidelines [14].

## Results

### Disease Model

**Figure 1:**
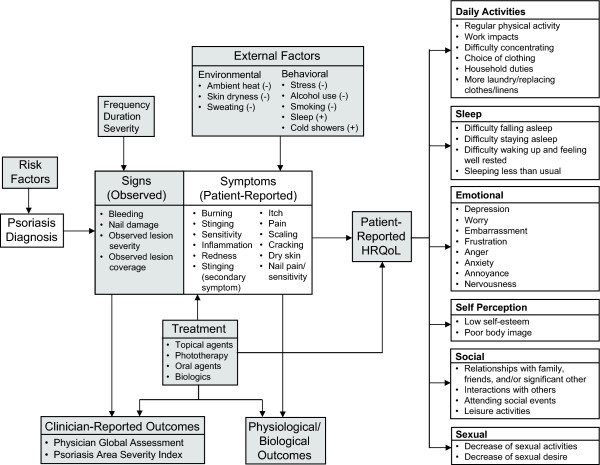
**Disease model of psoriasis**. The model illustrates the relationship among symptoms of psoriasis and their impacts on patients' everyday lives. Shaded boxes represent aspects of psoriasis included in the disease model for comprehensiveness, but were not evaluated as a part of this study.

The disease model (Figure 1) depicts a broad, big-picture view of issues related to psoriasis and the hypothesized inter-relationships. The major components of the disease model include: risk factors for psoriasis, psoriasis diagnosis, signs, symptoms, external factors, treatment, patient-reported HRQoL outcomes, clinician-reported outcomes, and physiological/biological outcomes. In this disease model, each box is linked, either directly or indirectly, to every other box. The arrows in the model illustrate direct linkage between different aspects of the disease and treatment. Because every box in the disease model is linked in varying degrees to every other box, it is impracticable to generate a schematic including all possible relationships among the different aspects of psoriasis; consequently this disease model includes the relationships of primary importance. Our analyses focused on the impact of patient-reported symptoms (center of the model) on the patient-reported HRQoL domains most proximal to the patients' experiences (right side of the model).

The disease model begins with a diagnosis of psoriasis. The signs and symptoms of the disease are determined upon presentation to the physician and subsequent diagnosis. How these signs and symptoms are interpreted by the patient and the clinician is dependent upon their frequency, duration, and severity. Important additions to the disease model based on patient feedback included symptoms (skin cracking and dry skin), daily activities (choice of clothing, work impacts, household duties, and more laundry/replacing clothes/linens), sleep impact (difficulty waking up and feeling well rested; sleeping less than usual), emotions (embarrassment, frustration, anger, anxiety, annoyance, and nervousness), and social impact (interaction with others, attending social events, leisure activities, and sexuality).

### Physician Interviews

The most frequently mentioned symptoms reported by the clinicians were itch (n = 5), psoriatic arthritis (or "joint pains"; n = 4), soreness or pain (n = 3), and flaking (n = 4). In terms of importance, the symptom of itch was rated a 10 by 3 clinicians and an 8 and a 7 by the 2 remaining clinicians, with 10 indicating the highest rank for importance. Three clinicians rated itch as the most bothersome symptom, though one of those clinicians rated itch, pain, burning, stinging, and flaking as equally bothersome symptoms to patients.

Exemplary quotes from clinician interviews regarding itch are presented in Table 1. Clinicians reported the location of itch was most commonly the scalp, anterior legs, the shins, groin, armpits, and also the back. Itch was found to be mostly associated with lesions on the body surface, though not exclusively. Clinicians also reported challenges to treating and managing itch symptoms, as there are no specific oral anti-itch medications and topical therapies lose effectiveness over time.

**Table 1:** Select Comments Regarding Itch Symptoms from Clinician Interviews

**Code**	**Clinician Comments**
**Assessment of Severity**	"By how much the patient describes the itch. Like, if they say, it wakes me up at night, or it keeps me up at night, or it's relentless, or it drives me crazy, I know the itch is severe. Plus, you know, I might ask the patient to grade it on a scale of one to ten, how bad is your itch, with ten being the worst, one is being pretty mild or not present. (...) Oh, just the one to ten scale, that's the only systematic way I can grade itch numerically." "Well, I don't have a scale typically in the clinic. There is no numeric scale that I use, but I go by whether it keeps them up at night, or not. That's typically the threshold. The severity of itching really keeps a patient aware at night, and that's about it. I know that's very subjective, but I don't use anything more quantifiable than itch."
**Itch**	"In psoriasis it's less than it is for atopic dermatitis. It doesn't usually keep them up at night, but it is, in some people, a symptom that is annoying, rather than disabling." "If they're able to sleep at night, or it disables them during the daytime, if they've got to stop what they're doing to scratch. I also – well, I look at their skin to see if there are scratch marks, and if there's other accompanying signs of what we call excoriations, where they're digging at their skin, scratching and digging at their skin." "Itch is a very bothersome symptom for psoriasis and other skin conditions such as eczema. And itch can be more problematic than pain. Itch is constant. Itch is something patients are much more aware of, and they're always scratching themselves to the point where they start to bleed, and their skin gets infected. And it's usually worse at nighttime, because patients are more aware about their body, as opposed to daytime, where people are more – you know, they're more busy at work and things. That's when then get home, then they start to itch more. But I would say itch by far is the number one factor that drives patients crazy."
**Relevance of itch**	"I think it's highly relevant, because it's probably the most day in, day out thing that a lot of people face in that the itching is – comes to the top of forefront of symptomology, whether it's itching in your scalp, or itching on the patches of psoriasis. And it's again probably one of the single most bothersome things, other than the fact that it's there."
**Location**	"Scalp – wherever the plaques are, but scalp drives them crazy. Anywhere on the body." "Is the itch from lesions? Usually, yes." "Commonly hands, knees, ankles." "Their scalp and their lower legs. It can be where there's no lesions in the scalp, but in the lower legs there usually some evidence of dry, scaling skin with some scratch marks."
**Treatment/management challenges**	"Like if the treatment works, that's great, but if it doesn't work, then we've got to keep adding things to help control the itch, whether it be a pill for itch that they take at nighttime, like an antihistamine, or using various creams to keep applying to control the itch... I would probably say maybe 60% of the time the itch can be controlled with one treatment, whether it be the shots or light therapy. And then 40% of the time it's not adequate, it's got to add in something else, like a topical cream, to control the itch." "Particular challenges (related to the treatment or management of the itch)? Again, I think it's related to treating the underlying psoriasis itself." "Yes, there's no specific oral anti-itch medication. And some of the topical anti-itch medications are not always effective for long periods of time over large areas of the skin. Effective or feasible. If they have total body involvement, it's hard to – lotions, anti-itch lotions all over their body."

### Patient Focus Groups

A total of 39 patients participated in the focus groups (Table 2). The age and gender distribution of the patients who participated in the study were representative of the psoriasis population, although the group of patients with severe disease had a slightly lower percentage of women (55%) than the group with mild disease (63%). Also, the mean age was lower in the group with mild disease (36 years) than in the group with severe disease (45 years).

**Table 2:** Demography, Disease Diagnoses, and Health Status of Participants in Patient Focus Groups^a^

	**Focus Groups**	
	
	**Patients with Mild Psoriasis (N = 8)**	**Patients with Severe Psoriasis (N = 31)**
Female sex, n (%)	5 (63)	17 (55)
Age, mean years (range)	36 (20 – 74)	45 (22 – 66)
Race/Ethnicity, n (%)		
White	5 (63)	24 (77)
Black/African American	0	4 (13)
Hispanic	1 (13)	1 (3)
Mexican-American	1 (13)	0
Arabic	1 (13)	0
Spanish	0	1 (3)
Disease Diagnosis, n (%)		
Psoriasis	8 (100)	29 (94)^b^
Co-existing Psoriatic arthritis	0	1 (3)
Heath Status, n (%)		
Excellent	1 (13)	3 (10)
Very good	4 (50)	9 (29)
Good	3 (38)	13 (42)
Fair	0	4 (13)
Poor	0	2 (7)

^a^Data not available for all participants for all characteristics so columns may not add up to 100%.

^b^Two patients did not fill out a page of the demographic form that included diagnosis of psoriasis. The clinicians for these patients confirmed the diagnosis of psoriasis.

N = total number of patients; n = number of patients for whom data are available

Exemplary quotes from the patient focus groups regarding the importance of itch in their everyday lives are presented in Table 3. When asked about the impact of psoriasis, patients with severe disease reported that itch symptoms affected concentration (n = 3), and regular physical activity (n = 3). All patients with severe disease reported that itching or scratching their psoriasis impacted their sleep quality. Five patients reported missing days at work or school because of itch symptoms.

**Table 3:** Select Comments Regarding Itch Symptoms from Patient Focus Groups

**Code/domain**	**Patient Comment (Severity of Psoriasis)**
**Symptoms/itch**	"You itch a lot. Scratch a lot, I mean." (Severe) "Scratch to relieve." (Severe) "It will spread over my whole body, the itching. I'll get scabs on my knees, on my elbows that (...) like yours (...) are very, very bad, and the more you scratch it, the worse it gets. (...) it just spreads." (Severe) "The worst is, for me, is just the itching. The itch and the dry." (Severe) "My itching, it gets inflamed. (...) It spreads – (...) And the more you scratch – you're scratching – (...) Mainly the itching." (Mild) "Because I'm scratching everywhere, people not knowing the reason why I'm scratching." (Mild)
**Change of psoriasis symptoms due to anxiety/worsen**	"So that's the worst, that's when I start to get really anxious. And it, like I said, it can change over the course of a day. When I know I got to start getting ready for work, that puts me in a whole other frame of mind and I will notice that I'm itching a little bit more and or maybe my hands are not as calm as they are on my days off." (Severe)
**Change of psoriasis symptoms due to stress/worsen**	"Mine only itches when I'm under stress." (Severe) "...I'll be thinking about it way too much, and then I'll start getting – affecting my skin, because the stress will make it outbreak, and then the outbreak, I'll want to itch, and just scratch..." (Severe)
**Change of psoriasis symptoms over day/varies**	"Yeah, for me it changes a lot too... Towards the end of the night is when it's usually more itchy for me." (Severe)
**Change of psoriasis symptoms over week/varies**	"...and then Sunday will hit, and then that's it. Then I'll start itching again, so it's like – and Mondays are so busy at work. And like towards Wednesday – that's – I don't know. It's like Monday and Wednesday. Those two days that – I don't know why those – hate those days. (Severe) "The itching to me varies. (...) Just sometimes I don't even think about it and other times it just, boom, it's just itching." (Severe)
**Impact on daily activities/difficulty concentrating**	"You lose concentration, because you want to scratch and (...) really want to itch this, but you don't want to itch it in front of somebody, and so you trail off to what you were originally helping somebody with, if you're working." (Mild) "Lack of concentration, or itchy – if you're really over-itchy, sometimes it's hard to concentrate on something else other than that whole-body itch." (Severe)
**Impact on daily activities/choice of clothing**	"For some reason whenever I have anything that's 100% cotton I tend to itch more. It gets irritated more, so everything is based on clothing or cotton. I try to buy a certain type of clothes. So and that's what I got to wear all the time." (Severe)
**Impact on emotions/embarrassed**	"I'm in front of people all day long, and it's incredibly embarrassing to start bleeding in front of someone, or scratching uncontrollably when you're not even thinking about it." (Severe) "Mine is just more of embarrassment. When you're scratching and people see things coming off of you or on your clothing..." (Mild)
**Impact on sleep/difficulty falling asleep**	"Before you go to sleep yeah. (...) Because your itches." (Mild) "It's itching instead of falling asleep." (Severe) "It's falling asleep, when it's itching, and you – and then the minute you start scratching, it only makes it worse. But it's hard...You tell yourself not to scratch, but you think you're going to stop it, you know? And then you scratch it, it only makes it worse, and you want to scratch more, and scratch more. It's bad." (Severe)
**Impact on sleep/difficulty staying asleep**	"Well, no. I'm going to wake up, I'm itchy. I'm going to put some cortisone on, I'm going grease myself down – then I'm going to try and go back to bed." (Severe) "Just, I want to rip my skin off, because it wakes me up. It's like it's never-ending." (Severe)
**Miss days of school or work because of psoriasis/itch**	"Yeah, you go every week and you get shots to stop you from itching." (Severe) "And I had it on my feet really bad, and I actually missed several days of work... Well, it's the itching." (Severe) "I can't just go out there (...) taking the train to work, being around a bunch of people, and coming back home – the whole time I just want to scratch and itch..." (Severe)

Patients with mild disease also reported difficulty concentrating due to itching or scratching, as well as knowing other people could see their psoriasis on their body. Two patients with mild disease mentioned that psoriasis affected them at work because of scratching. One patient spontaneously reported experiencing difficulty falling asleep because of itch symptoms. When probed, patients with mild disease reported that their difficulty sleeping was often due to scratching caused by their psoriasis.

The majority of patients rated itch as the most important and troublesome symptom (Table 4). The rating of itch as the most troublesome symptom was highest in patients with mild disease. The rating of itch as the most severe symptom was higher in patients with severe disease (mean rating 8.1 with 48% of patients rating the highest score of 10) than in patients with mild disease (mean rating 5.8 with 0% of patients rating the highest score of 10).

**Table 4:** Importance, Severity, and Troublesomeness of Itch Symptoms Rated by Patients With Mild and Severe Psoriasis

	**Patients with Mild Disease (N = 8)**	**Patients with Severe Disease (N = 31)**	**All Patients (N = 39)**
Patients rating itch as the most important symptom^a^			
No. of patients, n	8	23	31
Mean rating	9.1	8.5	8.7
Patients rating a 10, %	88%	70%	74%
Patients rating itch as the most severe symptom^b^			
No. of patients, n	8	23	31
Mean rating	5.8	8.1	7.5
Patients rating a 10, %	0%	48%	35%
Patients rating itch as the most troublesome symptom^c^			
No. of patients, n	8	16	24
Mean rating	9.0	8.0	8.3
Patients rating a 10, %	63%	50%	54%

^a^Symptoms were rated from 1 (least important) to 10 (most important)

^b^Symptoms were rated from 1 (least severe) to 10 (most severe)

^c^Symptoms were rated from 1 (least troublesome) to 10 (most troublesome)

Concepts that were added to the disease model based on patient feedback and saturation grid analysis are shown in Table 5. Results of the saturation analysis showed that no new sub-concepts were mentioned by patients in the final focus group. Therefore, it was concluded that saturation was reached in the focus groups.

**Table 5:** Representative Spontaneous Responses From the Concept Elicitation Saturation Grid From Patient Focus Groups

	**Total Responses**	**FG 1 vs FG 2**	**FG 1–2 vs FG 3**	**FG 1–3 vs FG 4**	**FG 1–4 vs FG 5**
**Symptoms**					
Itch	19	4 vs 4	8 vs 3	11 vs 5	16 vs 3
Bleeding	13	3 vs 5	8 vs 1	9 vs 3	12 vs 1
Cracking	12	0 vs 5	5 vs 4	9 vs 3	12 vs 0
Scaling	12	2 vs 4	6 vs 4	10 vs 1	11 vs 1
Dry skin	6	1 vs 1	2 vs 1	3 vs 3	6 vs 0
**Impact on daily activities**					
Choice of clothing	17	2 vs 6	8 vs 2	10 vs 4	14 vs 3
Effects on work	9	3 vs 2	5 vs 0	5 vs 2	7 vs 2
More laundry/replacing clothes and linens	2	2 vs 0	2 vs 0	2 vs 0	2 vs 0
Household duties	1	0 vs 1	1 vs 0	1 vs 0	1 vs 0
**Impact on social life**					
Interaction with others	15	3 vs 5	8 vs 3	11 vs 3	14 vs 1
Attending social events	5	0 vs 2	2 vs 2	4 vs 0	4 vs 1
Leisure activities	4	2 vs 1	3 vs 0	3 vs 1	4 vs 0
**Impact on sleep**					
Sleeping less than usual	2	2 vs 0	2 vs 0	2 vs 0	2 vs 0
Difficulty waking up and feeling well rested	1	0 vs 1	1 vs 0	1 vs 0	1 vs 0
**Impact on emotions**					
Embarrassed	17	5 vs 0	5 vs 3	8 vs 4	12 vs 5
Annoyed	7	2 vs 2	4 vs 2	6 vs 1	7 vs 0
Frustrated	4	0 vs 3	3 vs 0	3 vs 0	3 vs 1
Anxious	2	0 vs 0	0 vs 0	0 vs 1	1 vs 1
Nervous	1	0 vs 0	0 vs 1	1 vs 0	1 vs 0
**Impact on sex**					
Sexual activities	5	2 vs 0	2 vs 1	3 vs 0	3 vs 2
Decreased sexual desire	2	2 vs 0	2 vs 0	2 vs 0	2 vs 0

FG = Focus group

## Discussion

The disease model of psoriasis developed here represents a global picture of the concepts and domains of psoriasis while providing support for patient-based assessments as endpoints in clinical trials. Itch was identified as a characteristic symptom of psoriasis in the literature review and included as one of the patient-reported symptoms of psoriasis in the disease model. The majority of physicians and patients indicated that itch was the most important symptom of psoriasis. Clinicians agreed in their interviews with the relevance of itch as an important symptom in psoriasis. Furthermore, physicians noted that itch can often be recalcitrant to traditional treatments. In assessing the three qualities of itch (importance, severity, and troublesomeness), patients with severe psoriasis rated itch as more severe on the 10-point visual analogue severity scale and were more likely to select the most severe anchor of 10 (48% with severe psoriasis compared with 0% of mild psoriasis).

Itch, or pruritus, has been shown to be one of the most embarrassing [15] and distressing [16] symptoms for patients with psoriasis. This diagnostic symptom of psoriasis has profound effects on HRQoL [2,17-22]. The affective dimension of itch (described by patients as "unbearable," "worrisome," bothersome," and "annoying"), but not the sensory dimension of itch (described by patients as "burning," "stinging," and "crawling like ants"), is a significant predictor of depression, distress, and sleep impairment in patients with psoriasis [20]. To date, the importance and relevance of itch as a symptom in psoriasis patients has not been systematically assessed. Itch therefore represents an important PRO in clinical trials that should be considered when assessing the efficacy of treatment. Our study was not designed to determine causality between components (eg. symptoms and HRQoL) of the disease model. Better measures of itch should provide further insight into the relationships between triggers and aggravating factors and itch, as well as the effects of itch on HRQoL.

The results of the focus groups in this study are consistent with data from other focus groups [19] and a questionnaire-based study [22] that have documented the importance of itch to patients with psoriasis. Also consistent with these previously reported studies were the patients' identification of stress as an aggravating factor for itch and the negative impact of itch on sleep [19,22]. The results of these studies confirm the link between the impact of psoriasis symptoms and HRQoL in patients with psoriasis, as demonstrated in the disease model of psoriasis.

PROs used in clinical trials can provide fundamental information from the patients' perspective about the symptoms of psoriasis and the subsequent impacts that symptoms have on patients' lives. PROs are also fundamental in evaluating treatments in clinical trials to support approval, develop labeling, and substantiate potential advertising claims from a regulatory perspective. The results of our study demonstrate that itch matters to patients and clinicians, and assessment of itch should be included as a PRO in clinical trials of drugs used to treat psoriasis. The instrument used to assess itch should be clinically meaningful and be validated for reliability and responsiveness [23,24]. Other components of the disease model are also important; however, many of the items related to HRQoL can be captured in existing measures, such as the DLQI, SF-36, and EQ-5D.

The disease model of psoriasis developed in this study, in addition to the data from the physician interviews and patient focus groups, establishes a framework for the use of a PRO based on itch in clinical trials of drugs to treat psoriasis, in accordance with the first component of PRO development as required by the U.S. FDA [10,24]. Additional steps are to adjust the conceptual framework and draft the PRO instrument; confirm the measurement model and assess other measurement properties; modify the instrument; and collect, analyze, and interpret data. In accordance with FDA guidelines [24], the disease model was constructed using data from an extensive review of the literature. Clinician interviews confirmed essential elements of the disease model. Patient focus groups were used to prioritize aspects of psoriasis that are most relevant to patients, including the effects of itch on patients' everyday lives.

A limitation of this study was the nonrandom convenience sampling of both physicians and patients, which may not be representative of all dermatologists or patients with psoriasis. Qualitative research by its very nature requires small sample sizes, limiting the generalizability of the findings. Additionally, this study focused on symptoms of psoriasis; other domains in the disease model are equally important in understanding the impact of psoriasis on patients' lives but were outside the scope of these analyses.

Despite the small sample size, the entry criteria for participation in the study were similar to those used in clinical trials, increasing the likelihood of generalizability of our findings to other patients with psoriasis. All of the patients had physician-confirmed disease and represented the spectrum of disease severity in clinical practice. Because saturation was reached in the grid analysis, the sample size was adequate to meet our objective of achieving a better understanding of the impact of psoriasis on patients' lives.

Our results suggest that improvements in itch will result in improvements in patients' HRQoL. An anti-psoriatic drug that improves the symptoms of itch could therefore support a claim that the drug also improves HRQoL. The disease model reinforces the importance of measuring the components of disease as well as the patients' perspective within the clinical trial setting. In addition to the visual analog scale of itch severity frequently used in clinical trials, our study supports the development of itch questionnaires, such as the one developed by Yosipovitch et al [25], to fully assess the impact of itch on HRQoL in patients with psoriasis.

## Conclusion

These analyses enhanced our understanding of the impact of psoriasis symptoms on patients' lives, and suggest that itch is one of the most important symptoms of psoriasis, contributing to diminished HRQoL in patients with both mild and severe disease. Our results indicate a need for assessments of itch as well as skin lesions in clinical practice, and that itch should be considered as an endpoint in studies assessing the impact of disease and/or treatment in patients with psoriasis.

## Abbreviations

BSA: Body surface area; HRQoL: Health-related quality of life; IRB: Independent review board; PASI: Psoriasis Area and Severity Index; PRO: Patient-reported outcome.

## Competing interests

DG and DJH are compensated employees and shareholders in Amgen Inc. MSB has received research funding from Amgen Inc.

## Authors' contributions

DG made substantial contributions to the conception and design of the study and the interpretation of the data. MSB made substantial contributions to the conception and design of the study; and acquisition, analysis and interpretation of the data. DJH made substantial contributions to the interpretation of the data. All authors were involved in drafting the manuscript and revising it critically for important intellectual content, and have given final approval of the version to be published.
